# Simulation of *S*-Entropy Production during the Transport of Non-Electrolyte Solutions in the Double-Membrane System

**DOI:** 10.3390/e22040463

**Published:** 2020-04-18

**Authors:** Andrzej Ślęzak, Wioletta M. Bajdur, Kornelia M. Batko, Radomir Šcurek

**Affiliations:** 1 Department of Innovation and Safety Management Systems, Technical University of Czestochowa, 42200 Czestochowa, Poland; aslezak52@gmail.com; 2 Department of Business Informatics, University of Economics, 40287 Katowice, Poland; 3 Department of Security Services, Faculty of Safety Engineering, VSB-Ostrava, 70030 Ostrava, Czech Republic; radomir.scurek@vsb.cz

**Keywords:** membrane transport, Kedem–Katchalsky equations, double-membrane system, nonlinear model equations, *S*-entropy production

## Abstract

Using the classical Kedem–Katchalsky’ membrane transport theory, a mathematical model was developed and the original concentration volume flux (*J_v_*), solute flux (*J_s_*) characteristics, and *S*-entropy production by *J_v_*, $\left({\left(\psi_{S}\right)}_{J_{v}}\right)$ and by *J_s_* $\left({\left(\psi_{S}\right)}_{J_{s}}\right)$ in a double-membrane system were simulated. In this system, M_1_ and M_r_ membranes separated the *l*, *m*, and *r* compartments containing homogeneous solutions of one non-electrolytic substance. The compartment *m* consists of the infinitesimal layer of solution and its volume fulfills the condition *V_m_* → 0. The volume of compartments *l* and *r* fulfills the condition *V_l_* = *V_r_* → ∞. At the initial moment, the concentrations of the solution in the cell satisfy the condition *C_l_* < *C_m_* < *C_r_*. Based on this model, for fixed values of transport parameters of membranes (i.e., the reflection (*σ_l_*, *σ_r_*), hydraulic permeability (*L_pl_*, *L_pr_*), and solute permeability (*ω_l_*, *ω_r_*) coefficients), the original dependencies *C_m_* = *f*(*C_l_* − *C_r_*), *J_v_* = *f*(*C_l_* − *C_r_*), *J_s_* = *f*(*C_l_* − *C_r_*), ${\left(\Psi_{S}\right)}_{J_{v}}$ = *f*(*C_l_* − *C_r_*), ${\left(\Psi_{S}\right)}_{J_{s}}$ = *f*(*C_l_* − *C_r_*), *R_v_* = *f*(*C_l_* − *C_r_*), and *R_s_* = *f*(*C_l_* − *C_r_*) were calculated. Each of the obtained features was specially arranged as a pair of parabola, hyperbola, or other complex curves.

## 1. Introduction

One of the most important properties of each non-equilibrium thermodynamic system is the continuous production of *S*-entropy [1,2]. The temporal change in *S*-entropy is a consequence of the entropy exchange with the external environment (*φ_S_*) and the entropy production in the system (ψ*_S_*). This means that for irreversible processes occurring in open systems, the *S*-entropy rate of change (d*S*/d*t*) is the sum of the rate of entropy exchange with the external environment (*φ_S_* = *d_e_S*/*dt* < 0 or *d_e_S*/*dt* > 0) and the rate of entropy production in the system as a result of irreversible processes (ψ *_S_* = *d_i_S*/*dt* > 0) [1,2,3]. The rate of entropy production can be expressed using the expression $\Psi_{S}=\sum_{i}J_{i}X_{i}$, where $J_{i}X_{i}$ is the product of the conjugate forces ($X_{i}$) and fluxes ($J_{i}$). Prigogine [4] showed that for systems far from equilibrium, in the area of applicability of the extended non-equilibrium thermodynamics (ENET), $d\psi_{s}/dt=d_{J}\psi_{S}/dt+d_{X}\psi_{S}/dt=$ where $d_{J}\psi_{S}/dt\equiv \sum_{i}J_{i}\left(dX_{i}/dt\right)$, $d_{X}\psi_{S}/dt\equiv \sum_{i}X_{i}\left(dJ_{i}/dt\right),$ and $d_{X}\psi_{S}/dt$ ≤ 0. Furthermore, $d_{J}\psi_{S}/dt$ ≤ 0, in the regime of applicability of linear non-equilibrium thermodynamics (LNET).

Membrane transport processes on the nano-, micro-, and macro-scale are the subject of interest in different areas of human activity in science, technology, and medicine [5,6,7,8,9]. One of the most important scientific achievements in this area is the double-membrane model proposed by Curran and McIntosh. This model requires the existence of two membranes (M_l_, M_r_) with various hydraulic permeability (*L_pl_*, *L_pr_*), reflection (*σ_l_*, *σ_r_*), and solute permeability (*ω_l_*, *ω_r_*) coefficients, arranged in series and separating the solutions with different concentrations (*C_l_*, *C_m_*, *C_r_*) [10]. Papers published over several years have been dedicated to the analysis of transport in the double-membrane cell in order to clarify certain biophysical aspects of the membrane transport of water and dissolved substances, both in biological and artificial systems [11,12,13,14,15,16,17,18,19]. Recently, a double-membrane transducer protector [8] and double-membrane triple-electrolyte redox flow battery design [9] have been developed based on the concept of the two-membrane system.

On the basis of the characteristics illustrating the impact of concentration, pressure, and voltage dependencies on the volume flow, solute flow, and electric current, it has been shown that transport, in compliance with the concentration gradient, iso-osmotic transport, and the passive transport (against the concentration gradient), is possible in this system [11,12]. In addition, it has been shown that the double-membrane system is characterized by rectifying and amplification properties (asymmetry current-voltage characteristics [13,14,15,16,17,18,19]) and asymmetry and amplification of the volume and solute fluxes and hydromechanics pressure, which is characteristic for biological systems [20,21,22,23].

In the present paper, with the use of the Curran–Kedem–Katchalsky method utilized in the following papers [10,11,12,13,14,15,16,17,20,21,22,23], a non-linear mathematical model of transport in the double-membrane osmotic-diffusive cell was developed. This cell contains two membranes (M_l_, M_r_) arranged in series and separating the compartments (*l*), (*m*), and (*r*), which contain the solutions of various concentrations, respectively, *C_l_*, *C_m_*, and *C_r_* (at the initial moment *C_l_* > *C_m_* > *C_r_* or *C_l_* < *C_m_* < *C_r_*). The volume of these compartments satisfies the conditions: *V_m_* → 0 and *V_l_* = *V_r_* → ∞. Transport properties of the membranes M_l_ and M_r_ are characterized by coefficients of hydraulic permeability (*L_pl_*, *L_pr_*), reflection (*σ_l_*, *σ_r_*), and solute permeability (*ω_l_*, *ω_r_*). In order to search for new transport properties of the double-membrane system on the basis of the mathematical model, the calculations of the concentration (*C_m_*), volume flux (*J_v_*), solute flux (*J_s_*), *S*-entropy produced by *J_v_*, $\left({\left(\psi_{S}\right)}_{J_{v}}\right)$ and by *J_s_* $\left({\left(\psi_{S}\right)}_{J_{s}}\right)$, and osmotic and diffusion resistances (*R_v_*, *R_s_*).

## 2. Theory

### 2.1. Membrane System

**Figure 1:**
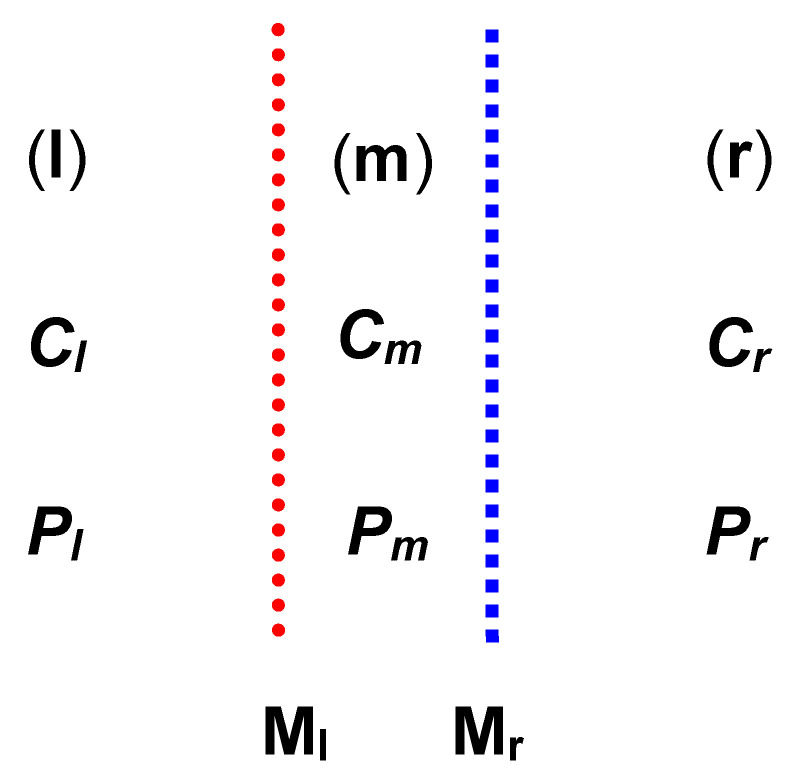
Model of double-membrane system: M_l_, M_r_ = membranes; *l*, *m*, *r* = compartments; *P_h_*, *P_m_*, *P_l_* = mechanical pressures; *C_l_*, *C_m_*, *C_r_* = concentrations of solutions.

Like in [7,8], let us consider the membrane system represented schematically in Figure 1. In this system, the compartments (*l*), (*m*), and (*r*), containing binary solutions of the same substance with the concentrations *C_l_*, *C_m_*, and *C_r_* (*C_l_* > *C_m_* > *C_r_*) are separated by electroneutral and selective membranes (M_l_, M_r_). The hydrostatic pressures in these compartments are denoted by *P_l_*, *P_m_*, and *P_r_* (*P_l_* > *P_m_* > *P_r_*) The membranes are characterized by the hydraulic permeability (*L_pl_*, *L_pr_*), reflections (*σ_l_*, *σ_r_*), and solute permeability (*ω_l_*, *ω_r_*). Compartment (*m*) consists of the infinitesimal layer of solution with the concentration *C_m_*. The volume of this compartment fulfills the condition *V_m_* → 0. The volumes of the compartments (*l*) and (*r*), containing solutions with the concentration *C_l_* and *C_r_*, fulfill the condition *V_l_* = *V_r_* → ∞.

The analysis of transport processes in this membrane system was based on the classical Kedem–Katchalsky model equations [15] using the Curran–Kedem–Katchalsky method [10,14,15]. Our starting point was the classical Kedem–Katchalsky model equations in binary and non-ionic solutions

(1) $$J_{s}=\omega \Delta \pi +J_{v}\left(1-\sigma \right)\bar{C}$$

(2) $$J_{v}=L_{p}\left(\Delta P-\sigma \Delta \pi \right)$$

where *J_s_* and *J_v_* are the solute and volume fluxes; *ω* is the solute permeability coefficient; *σ* is the reflection coefficient; *L_p_* is the hydraulic permeability coefficient; Δ*π* = *RT*Δ*C* is the osmotic pressure difference (*RT* is the product of the gas constant and absolute temperature); $\bar{C}$ is the average solution concentration in the membrane; and Δ*P* is the hydrostatic pressure difference. Equation (1) describes the solute flux, the first component of this equation, *J_sd_* = *ω*Δ*π*, describes the diffusive flux, and the second, *J_sa_* = *J_v_*(1 − *σ*) $\bar{C}$, is the advective flux. Then, Equation (2) describes the volume flux. It serves to note that the first component of this equation, *J_vh_* = *L_p_*Δ*P*, is the hydraulic volume flux, and the second one, *J_vo_* = *L_p_σ*Δ*π*, is the osmotic volume flux.

### 2.2. Model Equations

In order to describe the stationary volume flux in the membrane system shown in Figure 1, we considered Equation (1). The equations for membranes M_l_ and M_r_ are written in the following forms:

(3) $$J_{sl}=\omega_{l}RT\left(C_{l}-C_{m}\right)+J_{vl}\left(1-\sigma_{l}\right){\bar{C}}_{l}$$

(4) $$J_{sr}=\omega_{r}RT\left(C_{m}-C_{r}\right)+J_{vr}\left(1-\sigma_{r}\right){\bar{C}}_{r}$$

where ${\bar{C}}_{l}$ = (*C_l_* − *C_m_*)[ln(*C_l_C_m_*^−1^)]^−1^ ≈ $\frac{1}{2}$ (*C_l_* + *C_m_*), and ${\bar{C}}_{r}$ = (*C_m_* − *C_r_*)[ln(*C_m_C_r_*^−1^)]^−1^ ≈ $\frac{1}{2}$ (*C_m_* + *C_r_*).

In the steady state, the following conditions are fulfilled:

(5) $$J_{sl}=J_{sh}=J_{s}$$

(6) $$J_{vl}=J_{vr}=J_{v}$$

On the basis of Equations (3)–(6), we obtain

(7) $$C_{m}=\frac{RT\left(\omega_{l}C_{l}+\omega_{r}C_{r}\right)+\frac{1}{2}J_{v}\left[\left(1-\sigma_{l}\right)C_{l}-\left(1-\sigma_{r}\right)C_{r}\right]}{RT\left(\omega_{l}+\omega_{r}\right)+\frac{1}{2}J_{v}\left(\sigma_{l}-\sigma_{r}\right)}$$

In order to calculate *J_v_*, on the basis of Equation (2) for the membrane system presented in Figure 1, we can write

(8) $$J_{vl}=L_{pl}\left(P_{l}-P_{m}\right)-L_{pl}\sigma_{l}RT\left(C_{l}-C_{m}\right)$$

(9) $$J_{vr}=L_{pr}\left(P_{m}-P_{r}\right)-L_{pr}\sigma_{r}RT\left(C_{m}-C_{r}\right)$$

Combining Equations (6), (8), and (9), we obtain the equation describing hydrostatic pressure in the intermembrane compartment of the double-membrane system

(10) $$P_{m}=\frac{L_{pl}P_{l}+L_{pr}P_{r}-RT\left(L_{pl}\sigma_{l}C_{l}+L_{pr}\sigma_{r}C_{r}\right)+C_{m}RT\left(L_{pl}\sigma_{l}+L_{pr}\sigma_{r}\right)}{L_{pl}+L_{pr}}$$

Taking into consideration Equations (8) and (10), we obtain

(11) $$J_{v}=\frac{L_{pl}L_{pr}}{L_{pl}+L_{pr}}\left[\left(P_{l}-P_{r}\right)+RT\left(\sigma_{r}C_{r}-\sigma_{l}C_{l}\right)+RTC_{m}\left(\sigma_{l}-\sigma_{r}\right)\right]$$

Including Equation (11) into Equation (7), we derive

(12) $$\alpha_{1}{C_{m}}^{2}+\alpha_{2}C_{m}+\alpha_{3}=0$$

where *α*_1_ = *L_pl_L_pr_RT*(*σ_l_* − *σ_r_*)^2^; *α*_2_ = 2*RT*(*L_pl_* + *L_pr_*)(*ω_l_* + *ω_r_*) + *L_pl_L_pr_*(*σ_l_* − *σ_r_*)[(*P_l_* − *P_r_*) − *RT*(*C_l_* − *C_r_*)]; and *α*_3_ = −2*RT*(*L_pl_* + *L_pr_*)(*ω_l_C_l_* + *ω_r_C_r_*) − *L_pl_L_pr_*[(1 − *σ_l_*)*C_l_* − (1 − *σ_r_*)*C_r_*][(*P_l_* − *P_r_*) + *RT*(*σ_r_C_r_* − *σ_l_C_l_*)].

Hereby, we obtain the equation describing the solution concentration in the intermembrane compartment of the double-membrane system. Taking into consideration Equations (7) and (11), we obtain

(13) $$\beta_{1}{J_{v}}^{2}+\beta_{2}J_{v}+\beta_{3}=0$$

where *β*_1_ = 0.5(*σ_l_* − *σ_r_*); *β*_2_ = *RT*(*ω_l_* + *ω_r_*) − 0.5 *L_pl_L_pr_*(*L_pl_* + *L_pr_*)^−1^(*σ_l_* − *σ_r_*){(*P_l_* − *P_r_*) + *RT*[*C_l_*(1 − 2*σ_l_*) − *C_r_*(1 − 2*σ_r_*)]}, *β*_3_ = −*L_pl_L_pr_*(*L_pl_* + *L_pr_*)^−1^*RT*{(*P_l_* − *P_r_*)(*ω_l_* + *ω_r_*) − *RT*(*C_l_* − *C_r_*)(*σ_r_ω_l_* + *σ_l_ω_r_*)}.

Taking into consideration Equations (3), (5), and (7), we obtain

(14) $$J_{s}=\frac{\left(\gamma_{1}+\gamma_{2}{J_{v}}^{2}\right)\left(C_{l}-C_{r}\right)+\gamma_{3}J_{v}\left(C_{l}+C_{r}\right)}{\gamma_{4}+\gamma_{5}J_{v}}$$

where *γ*_1_ = (*RT*)^2^*ω_l_ω_r_*; *γ*_2_ = 0.5(1 − *σ_l_*)(1 − σ*_r_*); *γ*_3_ = 0.5*RT*[*ω_l_*(1 − *σ_r_*) + *ω_r_*(1 − *σ_l_*)]; *γ*_4_ = *RT*(*ω_l_* + *ω_r_*); and *γ*_5_ = 0.5(*σ_l_* − σ*_r_*).

On the basis of Equations (12)–(14), *C_m_*, *J_v_*, and *J_s_* can be calculated.

*J_v_* and *J_s_* can be used to calculate entropy production ($\psi_{S}$) in the double-membrane system, using the expression presented in a previous paper [24]. If there is only an osmotic pressure difference Δ*π* = *RT*Δ*C* in the double-membrane system, this expression can be written as

(15) $$\psi_{S}={\left(\psi_{S}\right)}_{J_{v}}+{\left(\psi_{S}\right)}_{J_{s}}=RJ_{v}\Delta C+RJ_{s}\frac{\Delta C}{\bar{C}}$$

where ${\left(\psi_{S}\right)}_{J_{v}}$ is the *S*-entropy produced by *J_v_* and ${\left(\psi_{S}\right)}_{J_{s}}$ is the *S*-entropy produced by *J_s_*.

## 3. Results and Discussion

**Figure 2:**
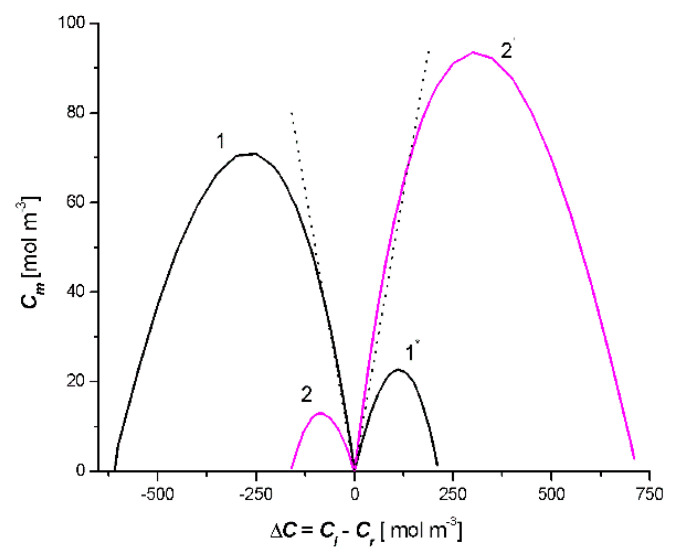
Dependencies *C_m_* = *f*(*C_l_* − *C_r_*) for the case *C_l_*/M_l_/*C_m_*/M_r_/*C_r_* (curves 1 and 1′) and *C_l_*/M_r_/*C_m_*/M_l_/*C_r_* (curves 2 and 2′).

**Figure 3:**
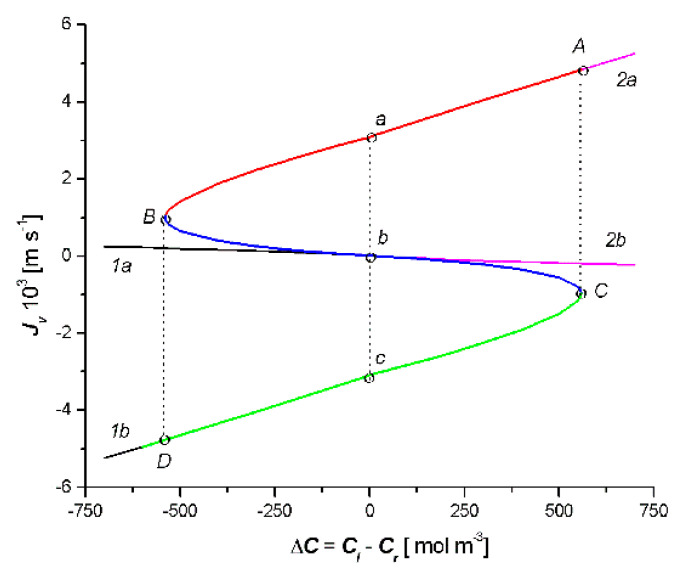
Dependencies *J_v_* = *f*(*C_l_* − *C_r_*) for the case *C_l_*/M_l_/*C_m_*/M_r_/*C_r_* (parabola with branches 1a and 1b) and *C_l_*/M_r_/*C_m_*/M_l_/*C_r_* (parabola with branches 2a and 2b).

**Figure 4:**
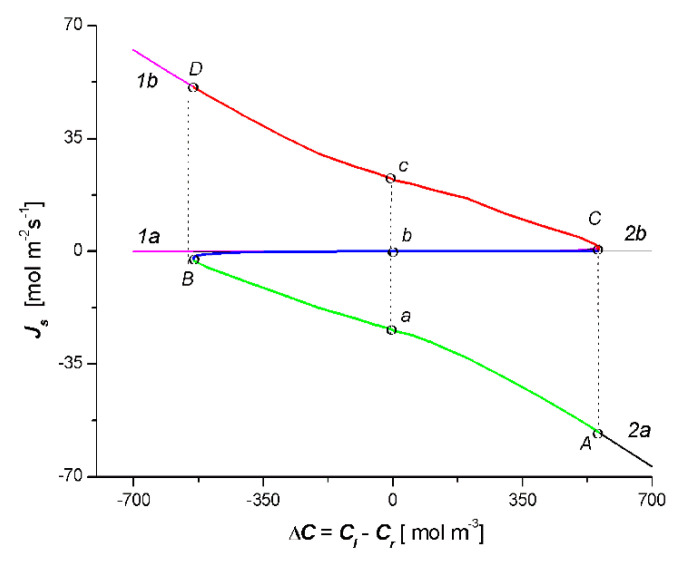
Dependencies *J_s_* = *f*(*C_h_* − *C_l_*) for the case *C_l_*/M_l_/*C_m_*/M_r_/*C_r_* (parabola with branches 1a and 1b) and *C_l_*/M_r_/*C_m_*/M_l_/*C_r_* (parabola with branches 2a and 2b).

The calculations of *C_m_* = *f*(*C_l_* − *C_r_*), *J_v_* = *f*(*C_l_* − *C_r_*), *J_s_*= *f*(*C_l_* − *C_r_*), ${\left(\psi_{S}\right)}_{J_{v}}$ *= f*(*C_l_* − *C_r_*), and ${\left(\psi_{S}\right)}_{J_{s}}$ *= f*(*C_l_* − *C_r_*) were obtained on the basis of Equations (12)–(15), respectively, for the fixed hydrostatic pressure difference in the double-membrane system Δ*P* = *P_l_* − *P_r_* = 13 kPa and for two cases: *C_l_*/M_l_/*C_m_*/M_r_/*C_r_* and *C_l_*/M_r_/*C_m_*/M_l_/*C_r_*. The concentration difference (Δ*C = C_l_* − *C_h_*) has changed in the range from −700 to +700 mol m^−3^. For the M_l_ and M_r_ membranes, the following values of transport parameters were used: *σ_l_* = 0.2, *σ_r_* = 0.1, *ω_l_* = 2 ×10^−8^ mol N^−1^s^−1^, *ω_r_* = 4 × 10^−8^ mol N^−1^s^−1^, *L_pl_* = 2 × 10^−9^ m^3^N^−1^s^−1^, and *L_pr_* = 4 × 10^−9^ m^3^N^−1^s^−1^. The obtained results of these calculations are shown in Figure 2, Figure 3, Figure 4, Figures 7 and 8.

In Figure 2, the results of the calculations *C_m_* = *f*(*C_l_* − *C_r_*) are shown. Parabolas 1 and 1’ were obtained for Case 1, and parabolas 2 and 2’ were obtained for Case 2. Figure 2 shows that parabola 1 crosses the concentration axis at the points *C_l_* − *C_r_* = −617.6 mol m^−3^ and *C_l_* − *C_r_* = 0, parabola 1’ at the points *C_l_* − *C_r_* = 0 and *C_l_* − *C_r_* = 213.2 mol m^−3^. The vertices of these parabolas have the following coordinates: *C_m_* = 71.7 mol m^−3^ and *C_l_* − *C_r_* = −275.7 mol m^−3^ (parabola 1) and *C_m_* = 22.8 mol m^−3^ and *C_l_* − *C_r_* = 110.3 mol m^−3^ (parabola 1′). In turn, parabola 2 intersected the concentration axis at the points *C_l_* − *C_r_* = −165.4 mol m^−3^ and *C_l_* − *C_r_* = 0; parabola 2′ at the points *C_l_* − *C_r_* = 0 and *C_l_* − *C_r_* = 720.6 mol m^−3^. The vertices of these parabolas had the following coordinates: *C_m_* = 13.1 mol m^−3^ and *C_l_* − *C_r_* = −84.5 mol m^−3^ (parabola 2) and *C_m_* = 90.3 mol m^−3^ and *C_l_* − *C_r_* = 305.1 mol m^−3^ (parabola 2′). The dotted lines illustrate the dependence of *C_m_* = *f*(*C_l_* − *C_r_*) for *C_m_* = 0.5(*C_l_* + *C_h_*). The results of the studies presented in Figure 2 indicate that in the double-membrane system, the solution accumulation effect of the intermembrane compartment of this system occurs for parabola 1 if *σ_l_* > *σ_r_*, *ω_l_* < *ω_r_*, *L_pl_* < *L_pr_* and −35.9 mol m^−3^ ≥ *C_l_* − *C_r_* < 0). For parabola 2′, if *σ_l_* < *σ_r_*, *ω_l_* > *ω_r_*, *L_pl_* > *L_pr_* and 0 < *C_l_* − *C_r_* ≤ 110.3 mol m^−3^). For *C_l_* − *C_r_* < −35.9 mol m^−3^ (parabola 1) and *C_l_* − *C_r_* > 110.3 mol m^−3^ (parabola 2′). For parabola 2 and 1’, the solution depletion effect of the intermembrane compartment of the double-membrane system occurs.

Figure 3 illustrates the results of the calculations of *J_v_* =*f*(*C_l_* − *C_r_*) based on Equation (13) for Cases 1 and 2. The results of the calculations are shown as the parabola with branches 1a and 1b and the parabola with branches 2a and 2b. The vertex of the first parabola (*C*) had the coordinates: *J_v_* = −0.92 × 10^−3^ m s^−1^ and *C_l_* − *C_r_* = 562.1 mol m^−3^. The vertex of the second parabola (*B*) had the coordinates: *J_v_* = 1 ×10^−3^ m s^−1^ and *C_l_* − *C_r_* = −546.4 mol m^−3^. The results of the studies presented in this figure show that the solution of Equation (13) is a pair of parabolas with the common point *J_v_* = 0 and *C_l_* − *C_r_* = 0.

Figure 4 presents the results of the calculation of *J_s_* = *f*(*C_l_* − *C_r_*) based on Equation (14) for the two cases. The obtained results of the calculations are presented as parabolas with branches 1a and 1b and parabola with branches 2a and 2b. The vertex of the first parabola (*C*) had the coordinates: *J_s_* = 2.4 mol m^−2^s^−1^ and *C_l_* − *C_r_* = 558.6 mol m^−3^ while the vertex of the second parabola (*B*) − *J_s_* = −2.4 mol m^−2^s^−1^ and *C_l_* − *C_r_* = −535.1 mol m^−3^. The results of the studies presented in Figure 4 show that the solution of Equation (14), similar to Equation (13), is a pair of parabolas with a common point, *J_s_* = 0 and *C_l_* − *C_r_* = 0.

We performed the procedure involving the omission of the fragments of the parabolas, which are shown in Figure 3 and Figure 4. If we leave branches 1b and 2a, section Cb of branch 1a of parabola 1 and section Bb of branch 2b, which are shown in Figure 3, we obtain the characteristic *J_v_* = *f*(*C_l_* − *C_r_*) of the *S* type. Following this procedure for the relation *J_s_* = *f*(*C_l_* − *C_r_*) (i.e., if we leave branches 1b and 2a, section Cb of branch 2b, and section Bb of branch 1a), which is shown in Figure 4, we obtain the characteristic *J_s_* = *f*(*C_l_* − *C_r_*) of the reversed letter S type. The curve, which is shown in Figure 3, illustrates the dependence of *J_v_* on the value of the control parameter Δ*C* = *C_l_* − *C_r_*, when the set value of the parameter Δ*C*_0_ = 0 corresponds to three stationary states of *J_va_* = 3.17 × 10^−3^ m s^−1^, *J_vb_* = 0, and *J_vc_* = 3.0 × 10^−3^ m s^−1^, respectively. Stable states located on the AB and CD sections of the curve were stable and the states located on the CB section were unstable. When the bifurcating values Δ*C*_1_ = −546.4 mol m^−3^ and Δ*C*_2_ = 562.1 mol m^−3^ were reached, the step transitions CA and BD appeared at the extreme points C and B of the curve, so the unstable states in the BC section never actually occur in real systems [25].

From the curves shown in Figure 3, it follows that for branch 1a in the area Δ*C* = *C_l_* − *C_r_* < 0, *J_v_* > 0 and for branch 2b in the area Δ*C* = *C_l_* − *C_r_* > 0, *J_v_* < 0. Similarly, for segments Bb of curve 2b and Ba of curve 2a, Δ*C* = *C_l_* − *C_r_* < 0, *J_v_* > 0 and for segments Cb of curve 1a and Ca of branch 1b: Δ*C* = *C_l_* − *C_r_* > 0, *J_v_* < 0. This means that, in these ranges of Δ*C* = *C_l_* − *C_r_,* osmotic transport occurs against the concentration gradient, furthermore, in areas where osmotic transport occurs, despite the concentration gradient *R_v_* < 0 (see Figure 7).

In turn, the curve presented in Figure 4 illustrates the dependence of *J_s_* on the value of the control parameter Δ*C* = *C_l_* − *C_r_*, when the set value of parameter Δ*C*_0_ = 0 corresponds to the three stationary states of *J_sc_* = 23 mol m^−2^s^−1^, *J_sb_* = 0, and *J_sa_* = −23 mol m^−2^s^−1^, respectively. Stationary states located on the AB and CD sections of the curve were stable and the stationary states located on the BC section were unstable. When the bifurcating values of Δ*C*_1_ = −535.1 mol m^−3^ and Δ*C*_2_ = 558.6 mol m^−3^ were reached, the step transitions of CD and BA appeared at the extreme points C and B of the curve, which is shown, so that unstable states on the BC section never actually occur in real systems [26]. In addition, from the curves that are shown in the Figure 4 results, for branch 1b in the area Δ*C* = *C_l_* − *C_r_* < 0, *J_s_* > 0 and for branch 2a in the area Δ*C* = *C_l_* − *C_r_* > 0, *J_s_* < 0. This means that in these ranges of Δ*C* = *C_l_* − *C_r_*, the diffusion transport takes place against the concentration gradient and in areas where diffusion transport occurs against the concentration gradient *R_s_* < 0 (see Figure 8).

The presented analysis shows that the double-membrane system, which is capable of functioning in one of two stable states, has the properties of a trigger. This means that there is a change from one stable state to another as a result of the change in the value of Δ*C* = *C_l_* − *C_r_* and the change in the triad value of the membrane parameters M_l_ (*L_pl_*, *σ_l_*, *ω_l_*) and M_r_ (*L_pr_*, *σ_r_*, *ω_r_*). Trigger properties play an important role in biological systems, defining the directional and stepping transition from one state to another (e.g., in the process of electrical impulse along the nerve fiber transmission or in cell differentiation processes) [25].

**Figure 5:**
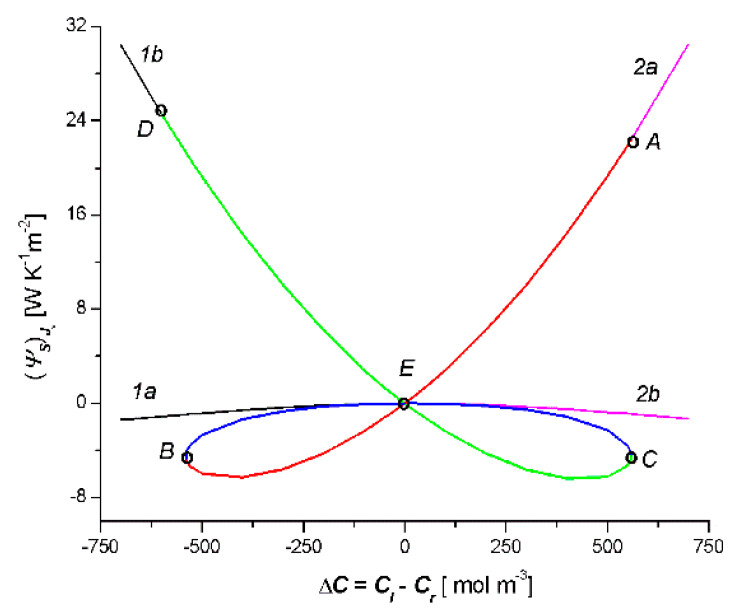
Dependencies ${\left(\Psi_{s}\right)}_{J_{v}}$ = *f*(*C_l_* − *C_r_*) for the case *C_l_*/M_l_/*C_m_*/M_r_/*C_r_* (curves 1a and 1b) and *C_l_*/M_r_/*C_m_*/M_l_/*C_r_* (curves 2a and 2b).

Figure 5 presents the results of the calculations ${\left(\psi_{S}\right)}_{J_{v}}$ (S-entropy produced by *J_v_*) based on Equation (15). This equation shows that in order to calculate ${\left(\psi_{S}\right)}_{J_{v}}$ we need to create the product of the universal gas constant (*R* = 8.31 J mol^−1^K^−1^), the results of the calculations *J_v_* (presented in Figure 3) and Δ*C* = *C_l_* − *C_r_*. Figure 5 shows that ${\left(\psi_{S}\right)}_{J_{v}}$ = *f*(*C_l_* − *C_r_*) is a combination of two curves 1a1b and 2a2b (two crossed bows in the shape of an inverted V), which intersect at the point with the coordinates ${\left(\psi_{S}\right)}_{J_{v}}$ = 0 and Δ*C* = 0. It should be noted that the colors of the elements of these curves correspond to the elements of the curves shown in Figure 3: the AB segment in Figure 4 corresponds to the AB segment in Figure 3, the BC segment in Figure 4 corresponds to the BC segment in Figure 3, and the CD segment in Figure 4 corresponds to the CD segment in Figure 3. Simultaneously, the sign ${\left(\psi_{S}\right)}_{J_{v}}$ depends on the sign *J_v_* and the sign Δ*C* = *C_l_* − *C_r_*. The comparison of Figure 5 and Figure 3 shows that for segment AE, the relations Δ*C* > 0 and *J_v_* > 0 were met; for segments *EB* and *BE*, Δ*C* < 0 and *J_v_* > 0; for sections *EC* and *CE*, Δ*C* > 0 and *J_v_* < 0; and for the segment *ED*, Δ*C* < 0 and *J_v_* < 0.

**Figure 6:**
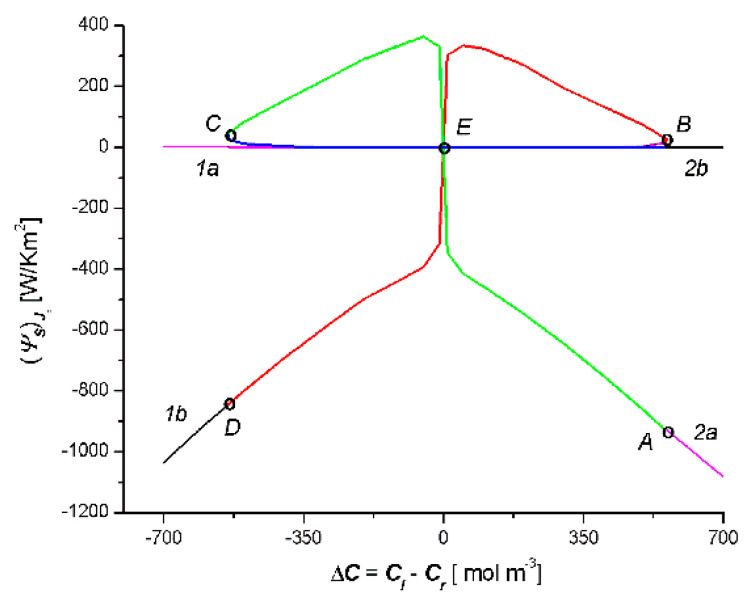
Dependencies ${\left(\Psi_{s}\right)}_{J_{s}}$ = *f*(*C_l_* − *C_r_*) for the case *C_l_*/M_l_/*C_m_*/M_r_/*C_r_* (curves 1a and 1b) and *C_l_*/M_r_/*C_m_*/M_l_/*C_r_* (curves 2a and 2b).

Figure 6 shows the results of the calculations ${\left(\psi_{S}\right)}_{J_{s}}$ (S-entropy produced by *J_s_*) based on Equation (15). This equation shows that, in order to calculate ${\left(\psi_{S}\right)}_{J_{s}}$, we need to create the product of the universal gas constant (*R* = 8.31 J mol^−1^K^−1^), the results of the calculations *J_s_* presented in Figure 4, Δ*C* = *C_l_* − *C_r_* and $\bar{C}$. Figure 6 shows that ${\left(\psi_{S}\right)}_{J_{s}}$ = *f*(*C_l_* − *C_r_*) is a combination of two curves 1a1b and 2a2b (bow in the shape of a jellyfish), which intersect at the point with the coordinates ${\left(\psi_{S}\right)}_{J_{s}}$ = 0 and Δ*C* = 0. It should be noted that the colors of the elements of these curves correspond to the elements of the curves shown in Figure 6: the AC segment in Figure 6 corresponds to the AC segment in Figure 4, the BD segment in Figure 6 corresponds to the BD segment in Figure 4, and the BC segment in Figure 6 corresponds to the BC segment in Figure 4. Simultaneously, the sign ${\left(\psi_{S}\right)}_{J_{s}}$ depends on the sign *J_s_* and the sign Δ*C* = *C_l_* − *C_r_*. The comparison of Figure 6 and Figure 4 shows that for segment AE, the relations Δ*C* > 0 and *J_s_* < 0 were met; for segments *EC* and *CE*, Δ*C* < 0 and *J_s_* < 0; for segments EB and BE, Δ*C* > 0 and *J_s_* > 0; and for the segment ED, Δ*C* < 0 and *J_s_* > 0.

Moreover, from Equation (15), it follows that ${\left(\psi_{S}\right)}_{J_{v}}$ > 0 when simultaneously *J_v_* > 0 and Δ*C* > 0 or when simultaneously *J_v_* < 0 and Δ*C* < 0. In turn ${\left(\psi_{S}\right)}_{J_{s}}$ > 0, when *J_s_* > 0 and Δ*C* > 0 or *J_s_* < 0 and Δ*C* < 0. When *J_v_* < 0 and Δ*C* > 0 or when *J_v_* < 0 and Δ*C* > 0, then ${\left(\psi_{S}\right)}_{J_{v}}$ < 0. In the case when *J_s_* < 0 and Δ*C* > 0 or when *J_s_* < 0 and Δ*C* > 0 simultaneously, then ${\left(\psi_{S}\right)}_{J_{s}}$ < 0. The relations ${\left(\psi_{S}\right)}_{J_{v}}$ < 0 and ${\left(\psi_{S}\right)}_{J_{s}}$ < 0 illustrate a deviation from the second law of thermodynamics for the membrane system. From this law, it follows that in the single-membrane system, thermodynamic fluxes reduce the value of stimuli (to which they are induced) and cause an equilibrium state. These thermodynamic fluxes are non-zero until ${\left(\psi_{S}\right)}_{J_{v}}$ > 0 and ${\left(\psi_{S}\right)}_{J_{s}}$ < 0. It seems that, in the double-membrane system, due to the occurrence of the phenomenon of accumulation or depletion of the substance in the inter-membrane compartment, cases where ${\left(\psi_{S}\right)}_{J_{v}}$ < 0 and ${\left(\psi_{S}\right)}_{J_{s}}$ < 0 are possible.

**Figure 7:**
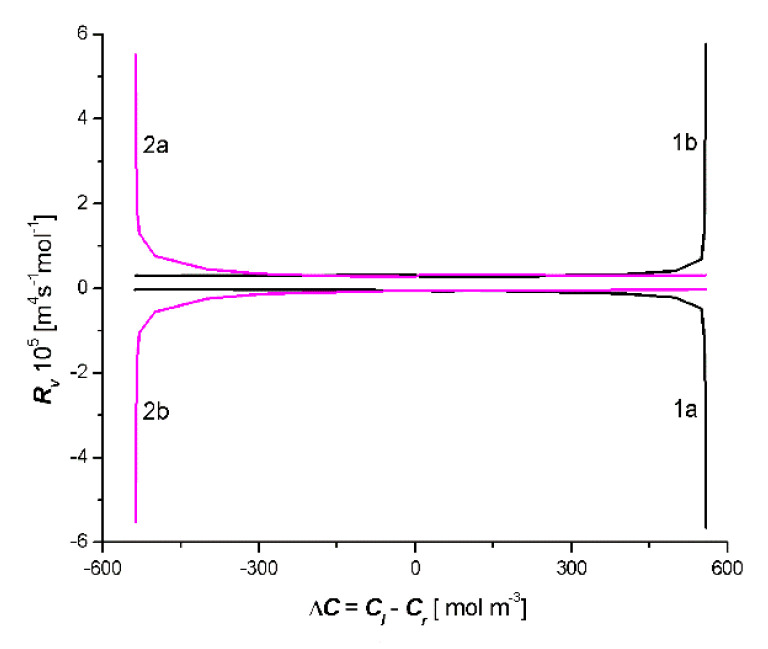
Dependencies *R_v_* = *f*(*C_l_* − *C_r_*) for the case *C_l_*/M_l_/*C_m_*/M_r_/*C_r_* (curves 1a and 1b) and *C_l_*/M_r_/*C_m_*/M_l_/*C_r_* (curves 2a and 2b).

**Figure 8:**
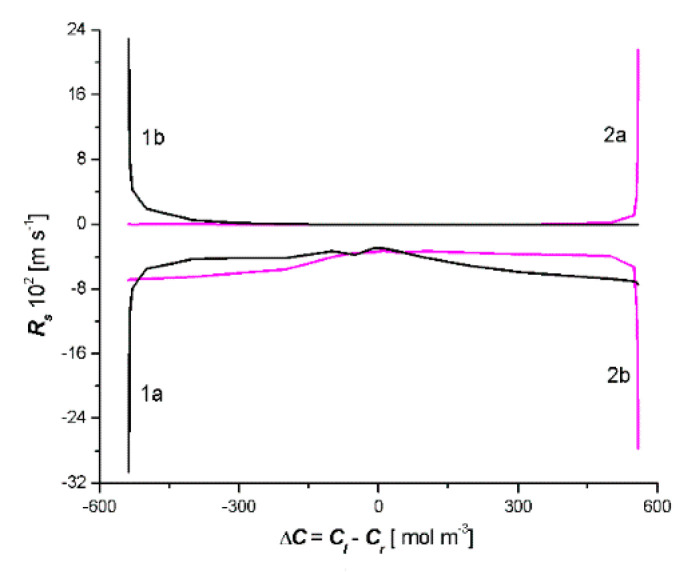
Dependencies *R_s_* = *f*(*C_h_* − *C_l_*) for the case *C_l_*/M_l_/*C_m_*/M_r_/*C_r_* (curves 1a and 1b) and *C_l_*/M_r_/*C_m_*/M_l_/*C_r_* (curves 2a and 2b).

Applying the results shown in Figure 7 and Figure 8, osmotic resistance (*R_v_*) and diffusion resistance (*R_s_*) were calculated using the following expressions:

(16) $$R_{v}=\frac{\Delta J_{v}}{\Delta \left(C_{l}-C_{r}\right)}$$

(17) $$R_{s}=\frac{\Delta J_{s}}{\Delta \left(C_{l}-C_{r}\right)}$$

The results of these calculations are shown in Figure 7 and Figure 8. It should be noted that curves 1a, 1b, 2a, and 2b (shown in Figure 7) were obtained from curves 1a, 1b, 2a, and 2b (presented in Figure 3). In turn, curves 1a, 1b, 2a, and 2b (shown in Figure 8) were obtained from curves 1a, 1b, 2a, and 2b (presented in Figure 4). Figure 5 shows that *R_v_* > 0 for curves 2a and 1b, furthermore, *R_v_* < 0 for curves 2b and 1a. Figure 8 shows that *R_s_* > 0 for curves 2b and 1a, furthermore, *R_s_* < 0 for curves 2a and 1b.

Negative resistance often determines the possibilities of their use of semiconductor components in electronics [26] and membrane systems in physiochemistry [27,28] and biophysics [29]. In membrane systems, negative resistance can be controlled by means of ion currents [29]. Therefore, the mechanism of negative resistance is the basis for the excitation of bio membranes [30].

All data were entered and calculated in Microsoft Excel 2016 and Origin Pro 2020.

## 4. Conclusions

These investigations showed that:

1. We created nonlinear model equations of the concentration in the inter-membrane compartment (*C_m_*), volume flux (*J_v_*), solute flux (*J_s_*), and *S*-entropy produced by *J_v_*, $\left({\left(\psi_{S}\right)}_{J_{v}}\right)$ and by *J_s_* $\left({\left(\psi_{S}\right)}_{J_{s}}\right)$ for binary homogeneous, non-electrolyte solutions. The created model equations, illustrated by Equations (12)–(15), consist of quadratic equations describing the concentration in the inter-membrane compartment (*C_m_*), volume flux (*J_v_*), and solute flux (*J_s_*) through the double-membrane system.
2. The double-membrane system, composed of two membranes (M_l_, M_r_), separates the compartments *l*, *m*, and *r* containing the homogeneous, non-electrolyte binary solutions. The compartment *m* consists of the infinitesimal layer of the solution and its volume fulfills the condition *V_m_* → 0. The volume of the compartments *l* and *r* fulfills the condition *V_l_* = *V_r_* → ∞. At the initial moment, the solution concentrations in the cell satisfy the condition *C_l_* < *C_m_* < *C_r_*.
3. Based on this model, for the fixed values of the reflection (*σ_l_*, *σ_r_*), hydraulic permeability (*L_pl_*, *L_pr_*), and solute permeability (*ω_l_*, *ω_r_*) coefficients, the dependencies *C_m_* = *f*(*C_l_* − *C_r_*), *J_v_* = *f*(*C_l_* − *C_r_*) and *J_s_* = *f*(*C_l_* − *C_r_*) were calculated. Each of the obtained characteristics was specifically arranged as a pair of parabolas.
4. The relationship ${\left(\psi_{S}\right)}_{J_{v}}$ = *f*(*C_l_* − *C_r_*) was a combination of two curves, 1a1b and 2a2b (two crossed bows in the shape of an inverted V), which intersected at the point with the coordinates ${\left(\psi_{S}\right)}_{J_{v}}$ = 0 and Δ*C* = 0. The sign ${\left(\psi_{S}\right)}_{J_{v}}$ was the consequence of the sign *J_v_* and Δ*C*: ${\left(\psi_{S}\right)}_{J_{v}}$ > 0 when simultaneously *J_v_* > 0 and Δ*C* > 0, or when simultaneously *J_v_* < 0 and Δ*C* < 0. If simultaneously *J_v_* < 0 and Δ*C* > 0 or when simultaneously *J_v_* < 0 and Δ*C* > 0, then ${\left(\psi_{S}\right)}_{J_{v}}$ < 0. In turn, the relationship ${\left(\psi_{S}\right)}_{J_{s}}$ = *f*(*C_l_* − *C_r_*) is a bow in the shape of a jellyfish. The sign ${\left(\psi_{S}\right)}_{J_{s}}$ was the consequence of the sign *J_s_* and Δ*C*: ${\left(\psi_{S}\right)}_{J_{s}}$ > 0 when simultaneously *J_s_* > 0 and Δ*C* > 0 or when simultaneously *J_s_* < 0 and Δ*C* < 0. If simultaneously *J_s_* < 0 and Δ*C* > 0 or when simultaneously *J_s_* < 0 and Δ*C* > 0, then ${\left(\psi_{S}\right)}_{J_{s}}$ < 0. The cases ${\left(\psi_{S}\right)}_{J_{v}}$ < 0 and ${\left(\psi_{S}\right)}_{J_{s}}$ < 0 indicate a deviation from the second law of thermodynamics caused by the phenomenon of the accumulation or depletion of the dissolved substance in the inter-membrane compartment of the double-membrane system.
5. In the solution concentration areas, where the relations were Δ*C* < 0, *J_v_* > 0 and *J_s_* > 0, Δ*C* > 0, *J_v_* < 0 and *J_s_* < 0, osmotic and diffusion transport (against the concentration gradient) occurred. In addition, in the areas where osmotic and diffusive transport took place (against the concentration gradient), osmotic and diffusion resistances (*R_v_*, *R_s_*) satisfied the conditions *R_v_* < 0 and *R_s_* < 0.
